# Determinants of frailty: the added value of assessing medication

**DOI:** 10.3389/fnagi.2015.00056

**Published:** 2015-04-21

**Authors:** Tiago Coelho, Constança Paúl, Robbert J. J. Gobbens, Lia Fernandes

**Affiliations:** ^1^Department of Occupational Therapy, School of Allied Health Technologies, Polytechnic Institute of Porto, Vila Nova de GaiaPortugal; ^2^The Research and Education Unit on Ageing, Institute of Biomedical Sciences Abel Salazar, University of Porto, PortoPortugal; ^3^Faculty of Health, Sports and Social Work, Inholland University of Applied Sciences, AmsterdamNetherlands; ^4^Zonnehuisgroep Amstelland, AmstelveenNetherlands; ^5^Center for Health Technology and Services Research (CINTESIS), Faculty of Medicine, University of Porto, PortoPortugal

**Keywords:** elderly, frailty, determinants, comorbidity, medication

## Abstract

This study aims to analyze which determinants predict frailty in general and each frailty domain (physical, psychological, and social), considering the integral conceptual model of frailty, and particularly to examine the contribution of medication in this prediction. A cross-sectional study was designed using a non-probabilistic sample of 252 community-dwelling elderly from three Portuguese cities. Frailty and determinants of frailty were assessed with the Tilburg Frailty Indicator. The amount and type of different daily-consumed medication were also examined. Hierarchical regression analysis were conducted. The mean age of the participants was 79.2 years (±7.3), and most of them were women (75.8%), widowed (55.6%) and with a low educational level (0–4 years: 63.9%). In this study, determinants explained 46% of the variance of total frailty, and 39.8, 25.3, and 27.7% of physical, psychological, and social frailty respectively. Age, gender, income, death of a loved one in the past year, lifestyle, satisfaction with living environment and self-reported comorbidity predicted total frailty, while each frailty domain was associated with a different set of determinants. The number of daily-consumed drugs was independently associated with physical frailty, and the consumption of medication for the cardiovascular system and for the blood and blood-forming organs explained part of the variance of total and physical frailty. The adverse effects of polymedication and its direct link with the level of comorbidities could explain the independent contribution of the amount of prescribed drugs to frailty prediction. On the other hand, findings in regard to medication type provide further evidence of the association of frailty with cardiovascular risk. In the present study, a significant part of frailty was predicted, and the different contributions of each determinant to frailty domains highlight the relevance of the integral model of frailty. The added value of a simple assessment of medication was considerable, and it should be taken into account for effective identification of frailty.

## Introduction

As age increases, physiological reserves inevitably decrease in multiple systems, and comorbidities become more prevalent (WHO, 1999). Nonetheless, chronological age is not a precise indicator of functional decline (Bergman et al., 2007). The changes that accompany aging depend on genetic and environmental factors, and are lifestyle and life event related (WHO, 1999). Therefore, while some may remain healthy and resilient in later life, others may become increasingly vulnerable to internal and external stressors. The latter refers to a state of frailty.

Frail individuals are at greater risk of clinically significant adverse outcomes such as hospitalization, institutionalization and mortality (Fried et al., 2004, 2009; Abellan van Kan et al., 2008). Although frailty is generally considered a clinical syndrome separate from the normal aging process, there are different perspectives about its definition (Hogan et al., 2003; Markle-Reid and Browne, 2003; Bergman et al., 2007; Sternberg et al., 2011). More traditional approaches to the concept describe frailty as an exclusively physical condition (presence of three or more of the following components: weight loss, low physical activity, exhaustion, slowed performance, and weakness; Fried et al., 2001), or as a result of the accumulation of multidimensional deficits (e.g., disabilities, symptoms, signs, diseases; Rockwood and Mitnitski, 2007). On the other hand, following the more current trends of frailty definition, the recently described integral conceptual model specified frailty as a dynamic pre-disability state that includes losses in physical, psychological and/or social domains (Gobbens et al., 2010a,b,c).

A broader definition of frailty also involves that the factors considered as underlying a state of increased vulnerability are beyond the decline of physiological reserve and comorbidity. In fact, according to the integral conceptual model of frailty, life course determinants such as sociodemographic characteristics and lifestyle, life event and environment-related factors can directly influence frailty, besides influencing the onset of diseases which can also lead to frailty (Gobbens et al., 2010c,d, 2012). From this standpoint, as multiple circumstances may impact the onset of frailty in older persons, researchers should focus on ascertaining which elements are associated with frailty in different contexts.

This study’s main objective was to analyze which determinants – described in the integral conceptual model of frailty – contribute to the prediction of frailty in general and of each frailty domain (physical, psychological, and social), in a sample of Portuguese community-dwelling individuals aged 65 years and over. Furthermore, the present study examined if a simple and objective measurement, such as assessing the number of daily-consumed medications, could help to explain frailty variance, after controlling for the effect of the determinants. It is hypothesized that a higher medication consumption is independently associated with increased frailty levels. In this context, the independent contribution of each type of daily-consumed drugs was also studied.

## Materials and Methods

### Study Design and Participants

A cross-sectional study was conducted with a non-probabilistic sample of 252 community-dwelling elderly (aged 65 years and over), in three northern Portuguese cities (Maia, Porto, and Vila Nova de Gaia).

Exclusion criteria were severe cognitive impairment (according to guidelines of the National Institute for Health and Care Excellence (NICE, 2011), participants with Mini Mental State Examination (Folstein et al., 1975) scores below 10 were excluded) and being unable to speak Portuguese.

Participants were interviewed in 16 local community institutions, such as social, recreation and day care centers, as well as senior universities. Trained researchers conducted the interviews from May to September 2013, using structured questionnaires. The study was approved by institutional review boards and by the ethics committee of the Ph.D. in Gerontology and Geriatrics (Institute of Biomedical Sciences Abel Salazar – University of Porto). All the participants gave their written informed consent before the interview.

### Measurements

Frailty and determinants of frailty were assessed with the Tilburg Frailty Indicator (TFI; Gobbens et al., 2010e), which is an operationalization of the integral conceptual model of frailty. This brief self-report questionnaire comprises two subscales (parts A and B). Part A is composed of 10 questions about determinants of frailty: sociodemographic characteristics (age, sex, marital status, nationality, level of education, and income); life events in the last year (death of a loved one, serious illness, serious illness in a loved one, divorce or end of an important relationship, traffic accident, and crime); assessment of how healthy the respondent’s lifestyle is; satisfaction with home living environment; and the presence of two or more chronic diseases. Part B measures frailty in three domains: physical (physical health, unexplained weight loss, difficulty in walking, difficulty in maintaining balance, hearing problems, vision problems, lack of strength in hands, and physical tiredness), psychological (cognition, depression and anxiety symptoms, and coping), and social (living alone, social relations and support). All items are rated dichotomously (0–1), with higher scores meaning higher frailty. Scores for each frailty domain and a total frailty score (0–15) are produced. The Portuguese version of TFI (Coelho et al., 2014) was used. This tool has a good internal consistency (KR-20 = 0.78) and test–retest reliability (*r* = 0.91) for total frailty score, and there is encouraging evidence regarding its construct and criterion validity (Coelho et al., 2014).

Medication was assessed in terms of the type and number of different daily-consumed drugs. In order to prevent recall bias, participants were previously asked to bring their medication or prescriptions to the interview. Based on the guidelines for ATC classification and DDD assignment (WHO, 2014), the following groups of medication were considered: cardiovascular system [e.g., diuretics and angiotensin-converting-enzyme (ACE) inhibitors], nervous system (e.g., psycholeptics and analgesics), metabolism (e.g., antidiabetics and mineral supplements), musculoskeletal system (e.g., anti-inflammatories and antirheumatics), digestive system (e.g., antacids and laxatives), blood and blood forming organs (e.g., antiplatelets and anticoagulants), respiratory system (e.g., bronchodilators and antihistamines), genitourinary system (e.g., antispasmodics and medicines for benign prostatic hyperplasia), endocrine system (e.g., corticosteroids and drugs for thyroid-related diseases), and other clinical conditions (e.g., infections and diseases of the sensory system).

Finally, measures of cognitive [MMSE (Folstein et al., 1975)], functional [Barthel Index (Mahoney and Barthel, 1965)/Lawton and Brody Scale (Lawton and Brody, 1969)] and nutritional status [Body Mass Index (BMI)] were used for the descriptive analysis of the sample.

### Statistical Analysis

Descriptive statistical analysis was performed using proportions and measures of central tendency and dispersion, according to the nature of the variables.

Linear regressions were conducted to ascertain how each determinant predicts frailty total score and scores per domain. Hierarchical multiple regression analysis were also performed, consisting mainly of five steps: in the first one, sociodemographic characteristics and life events were entered as predictors; second, assessment of lifestyle and satisfaction with living environment; third, self-reported comorbidity; fourth, number of daily-consumed drugs; and fifth, types of medication. In a secondary analysis, the MMSE score was inserted in a sixth step, in order to control for cognitive status.

As in previous studies (Gobbens et al., 2010d, 2012), life event “serious illness in the last year” was excluded from the analysis because it overlaps with comorbidity. Likewise, marital status was not considered for the prediction of total frailty and social frailty because it is closely linked with the TFI item “living alone.” Variables that revealed low frequencies (<5%) in the descriptive analysis were excluded in the regression models.

Two-tailed tests were used throughout all analyses and a *p*-value < 0.05 was considered statistically significant. All statistical analyses were performed with IBM SPSS Statistics 22.0 (SPSS, Inc., Chicago, IL, USA).

## Results

### Descriptive Analysis

The mean age of the participants was 79.2 years (±7.3), and they were mostly women (75.8%), widowed (55.6%), and with low education level (63.9%). The most common monthly household income was 251–500 euros (32.9%). The most shared life event (28.2%) was “serious illness in a loved one,” most described their lifestyle as healthy (54.4%), and were satisfied with their home living environment (79.0%). The majority of these elderly reported the presence of two or more chronic illnesses (53.2%), and the mean number of different daily-consumed medications was 5.3 (±3.1). The most frequent medications in this sample have been prescribed for the cardiovascular system (78.6%), nervous system (59.9%) and blood and blood forming organs (40.9%). The mean frailty total score was 6.0 (±3.4), and 2.9 (±2.2), 1.7 (±1.1), and 1.4 (±1.0) for the physical, psychological and social domains respectively. The mean MMSE score was 23.6 (±4.9), and regarding the Barthel Index and the Lawton and Brody Scale, the mean scores were 19.0 (±1.5) and 17.5 (±5.6), respectively. Finally, the mean BMI of the participants was 28.6 (±5.4). **Table 1** provides a more detailed description of the participants’ characteristics.

**Table 1:** Characteristics of the participants (*n* = 252) in regard to Determinants of frailty, frailty, medication.

Characteristics	*n* (%)
**Determinants of frailty (TFI part A)**
Age (years), mean ± SD	79.2 ± 7.3
65–74	68 (27.0)
75–84	116 (46.0)
≥85	68 (27.0)
Sex (women)	191 (75.8)
Nationality (Portuguese)	251 (99.6)
Marital status	
Married/living with partner	49 (19.4)
Unmarried	24 (9.5)
Separated/divorced	39 (15.5)
Widow/widower	140 (55.6)
Education (years), mean ± SD	4.4 ± 3.6
0	36 (14.3)
1–4	161 (63.9)
≥5	55 (21.9)
Monthly household income (euros)	
≤250	20 (7.9)
251–500	83 (32.9)
501–750	50 (19.8)
751–1000	44 (17.5)
1001–1500	25 (9.9)
1501–2000	22 (8.7)
≥2001	8 (3.2)
Life events	
Death of a loved one	55 (21.8)
Serious illness	56 (22.2)
Serious illness in a loved one	71 (28.2)
End of important relationship	8 (3.2)
Traffic accident	1 (0.4)
Crime	14 (5.6)
Lifestyle self-assessment	
Healthy	137 (54.4)
Not healthy, not unhealthy	92 (36.5)
Unhealthy	23 (9.1)
Satisfaction with living environment	199 (79.0)
Self-reported comorbidity	134 (53.2)
**Frailty (TFI part B)**
TFI total score (0–15), mean ± SD	6.0 ± 3.4
TFI physical domain score (0–8), mean ± SD	2.9 ± 2.2
TFI psychological domain score (0–4), mean ± SD	1.7 ± 1.1
TFI social domain score (0–3), mean ± SD	1.4 ± 1.0
**Medication**
Number of daily-consumed medication, mean ± SD	5.3 ± 3.1
Types of daily-consumed medication	
Cardiovascular system	198 (78.6)
Nervous system	151 (59.9)
Metabolism	74 (29.4)
Musculoskeletal system	60 (23.8)
Digestive system	93 (36.9)
Blood and blood forming organs	103 (40.9)
Respiratory system	29 (11.5)
Genitourinary system	19 (7.5)
Endocrine system	20 (7.9)
Other	23 (9.1)
**Cognitive status**
MMSE (0–30), mean ± SD	23.6 ± 4.9
**Functional status**
Barthel Index (0–20), mean ± SD	19.0 ± 1.5
Lawton and Brody Scale (0–23), mean ± SD	17.5 ± 5.6
**Nutritional status**
BMI (kg/m^2^), mean ± SD	28.6 ± 5.4

### Regression Analysis

First, due to the low percentage of non-Portuguese individuals, nationality was excluded from the regression analysis. Likewise, life events “divorce or end of important relationship” and “traffic accident” were left out due to the same reason. Also resulting from the descriptive analysis, the last two categories of income “1501–2000” and “≥2001” were regrouped in the single category “≥1501” before inclusion in the regression models. A dummy variable “cohabit” (“1” for married/living with partner and “0” for unmarried, separated/divorced and widow/widower) was created as an alternative to marital status. A dummy variable for sex was also created (“1” for women and “0” for men), and lifestyle was rated “1” for “healthy,” “2” for “not healthy, not unhealthy,” and “3” for “Unhealthy.” Preliminary analysis showed that the effects of education, income and lifestyle were linear, whereas the effects of age were both linear and quadratic. Consequently, age was squared and centered to allow the analysis of both effects on the regression models.

**Table 2** presents the effects of the determinants on TFI total score and their significance in the four steps of the hierarchical regression. The first one showed that age had a quadratic effect on frailty, with the youngest and oldest participants having less frailty. Women were, on average, frailer than men, as well as those who experienced the death of a loved one in the last year. On the other hand, as monthly income increases, frailty levels decrease. Education and life events “serious illness in a loved one” and “crime” had no effect on frailty. A total of 17.2% of frailty was predicted in the first step. In the second step, an additional 22.9% was predicted. Unhealthy lifestyle and dissatisfaction with living environment were associated with higher frailty. By including self-reported comorbidity in the third step, 5.9% of the variance of frailty was further predicted, with the presence of comorbidity being associated with a higher degree of frailty. By adding the amount of daily-consumed medication, an additional 2.5% of frailty was predicted, while the effect of age on frailty was no longer significant. As hypothesized, a higher number of medications was associated with higher frailty levels. However, after including the types of drugs in the regression model, the independent effect of the number of daily-consumed drugs on total frailty was no longer significant. In this last step, an additional 4.2% of frailty was predicted, with significant contributions of the variables concerning medications for the cardiovascular system and for the blood and blood forming organs. Frailty was lower in the participants that consumed drugs such as diuretics and ACE inhibitors, and higher for those who consumed drugs such as antiplatelets and anticoagulants.

**Table 2:** Results of hierarchical regression analysis on frailty.

Determinants	Model 1			Model 2			Model 3			Model 4			Model 5		
	*b*	95%CI	*r*	*b*	95%CI	*r*	*b*	95%CI	*r*	*b*	95%CI	*r*	*b*	95%CI	*r*
**Age**
Linear effect	0.02	-0.04; 0.08	0.03	0.02	-0.03; 0.07	0.05	0.04	-0.01; 0.09	0.08	0.04	-0.01; 0.09	0.07	0.04	-0.01; 0.09	0.07
Quadratic effect	-0.01**	-0.02; 0.00	0.16	-0.01*	-0.01; 0.00	-0.13	-0.01*	-0.01; 0.00	-0.10	-0.01	-0.01; 0.00	-0.08	0.00	-0.01; 0.00	-0.07
Sex (women vs. men)	1.41**	0.46; 2.35	0.17	1.25**	0.43; 2.06	0.16	0.95*	0.17; 1.74	0.11	1.03**	0.26; 1.80	0.12	0.84*	0.04; 1.64	0.09
Education	-0.11	-0.24; 0.01	-0.10	-0.09	-0.20; 0.02	-0.09	-0.06	-0.16; 0.04	-0.05	-0.03	-0.14; 0.07	-0.03	-0.06	-0.16; 0.05	-0.05
Monthly household income	-0.43**	-0.71; -0.15	-0.18	-0.35**	-0.59; -0.11	-0.15	-0.31**	-0.54; -0.08	-0.13	-0.32**	-0.55; -0.10	-0.13	-0.29*	-0.52; -0.07	-0.12
**Life events**
Death of a loved one	1.15*	0.15; 2.14	0.13	1.01*	0.16; 1.86	0.12	1.08**	0.27; 1.90	0.13	1.05**	0.26; 1.85	0.12	0.94*	0.14; 1.74	0.10
Serious illness in a loved one	0.30	-0.61; 1.22	0.04	0.20	-0.58; 0.99	0.03	0.15	-0.60; 0.89	0.02	0.07	-0.66; 0.80	0.01	0.21	-0.51; 0.043	0.03
Crime	0.25	-1.49; 2.00	0.02	0.31	-1.18; 1.80	0.02	0.06	-1.36; 1.48	0.00	-0.02	-1.41; 1.37	-0.00	-0.24	-1.62; 1.13	-0.02
Lifestyle				1.73***	1.18; 2.28	0.31	1.48***	0.95; 2.01	0.26	1.41***	0.90; 1.93	0.25	1.41***	0.89; 1.92	0.24
Satisfaction living environment				-2.35***	-3.22; -1.49	-0.27	-1.96***	-2.80; -1.12	-0.22	-2.01***	-2.83; -1.19	-0.22	-2.01***	-2.84; -1.19	-0.22
Self-reported comorbidity							1.82***	1.11; 2.52	0.24	1.39***	0.66; 2.12	0.17	1.66***	0.91; 2.41	0.20
Amount of drugs										0.20***	0.08; 0.31	0.16	0.17	-0.02; 0.37	0.08
**Types of medication**
Cardiovascular system													-1.18*	-2.11; -0.25	-0.11
Nervous system													0.53	-0.21; 1.27	0.06
Metabolism													0.54	-0.26; 1.34	0.06
Musculoskeletal system													0.44	-0.37; 1.25	0.05
Digestive system													-0.13	-0.91; 0.65	-0.01
Blood and blood forming organs													0.82*	0.08; 1.55	0.10
Respiratory system													-0.45	-1.52; 0.61	-0.04
Genitourinary system													-0.89	-2.23; 0.45	-0.06
Endocrine system													-0.07	-1.26; 1.13	0.00
Δ*R*^2^ (%; *p*-value)			17.2***			22.9***			5.9***			2.5***			4.2*

Regression coefficient (*b*), semi-partial correlation coefficient (*r*) and *p*-value for each determinant, and coefficient of determination change (Δ*R^*2*^*) and *p*-value for each model. *p < 0.05, **p < 0.01, ***p < 0.001.

In regard to physical frailty, a total of 51.3% of TFI physical domain score was predicted (step 1: Δ*R*^2^ = 14.2%; step 2: Δ*R*^2^ = 19.7%; step 3: Δ*R*^2^ = 5.9%; step 4: Δ*R*^2^ = 5.3%; step 5: Δ*R*^2^ = 6.2%). In the last model, physical frailty was associated with age (positive linear effect), death of a loved one in the last year, unhealthy lifestyle, dissatisfaction with living environment, self-reported comorbidity, higher amount of medications and, likewise to total frailty, non-consumption of drugs for the cardiovascular system and consumption of drugs for the blood and blood forming organs. The quadratic effect of age was no longer significant after adding lifestyle and satisfaction with living environment, whereas sex and education no longer contributed to physical frailty prediction after adding self-reported comorbidity. Income, serious illness in a loved one, crime, cohabitation and other types of medication had no effect on physical frailty.

Psychological frailty was significantly higher in women, in participants who had experienced the death of a loved one in the last year, had an unhealthy lifestyle, weren’t satisfied with living environment and reported comorbidity. The effect of education was only significant in the first step, whereas the contribution of age, income, cohabitation, the remainder life events and the number and type of medications was non-significant throughout all the steps. A total of 25.3% of TFI psychological domain was predicted in the first three models (step 1: Δ*R*^2^ = 10.8%; step 2: Δ*R*^2^ = 11.9%; step 3: Δ*R*^2^ = 2.6%).

Likewise, the variables concerning daily-consumed medication did not contribute to the prediction of social frailty. Remarkably, neither did self-reported comorbidity. A total of 27.7% was predicted in the first two steps (step 1: Δ*R*^2^ = 19.3%; step 2: Δ*R*^2^ = 8.4%). Social frailty was associated with age (quadratic effect), being female, higher levels of education, lower income, lifestyle and satisfaction with living environment.

Finally, the effect of the MMSE score on frailty in general and each frailty domain was non-significant, and its inclusion in the regression models (in a sixth step) did not influence the previously observed relationships between variables.

## Discussion

A significant proportion of frailty was predicted by life course determinants and by comorbidity. It was also possible to ascertain that each determinant played a different role in the prediction of frailty in general and in each frailty domain. This provides robust evidence to support the integral conceptual model of frailty (Gobbens et al., 2010c). On the other hand, the number of daily-consumed drugs was independently associated with physical frailty, and the consumption of medication for the cardiovascular system (e.g., antihypertensives) and for the blood and blood-forming organs (e.g., antithrombotics) explained part of the variance of total and physical frailty.

The observed effect of age on frailty was complex. As in other studies (Avila-Funes et al., 2008; Gobbens et al., 2010d; Collard et al., 2012), physical frailty was associated with increased age. This result was expected considering the physical toll of aging (Fried et al., 2009). However, total frailty was highest in participants aged between 75 and 84 years old, mainly because of the higher social frailty observed in this group. In fact, most of the participants who lived alone were included in this age group, possibly due to the fact that most of the younger participants still lived with their spouses, and that many older and widowed individuals lived with younger family members in order to receive the support needed to overcome their physical impairments. Nonetheless, the fact that age was no longer significant in frailty prediction after adding medication to the regression analysis, indicates that other determinants, including comorbidity, better explain the variance of frailty.

Similarly to previous research (Puts et al., 2005; Song et al., 2010; Collard et al., 2012), women were frailer than men. It has been shown that elderly men have a greater likelihood of dying suddenly, while women more often show a steady progressive decline, associated with an increase in morbidity (Puts et al., 2005). This fact can explain the present findings, including why the initial sex-based difference in physical frailty has disappeared after controlling for comorbidity.

As expected (Fried et al., 2001; Woo et al., 2005; Avila-Funes et al., 2008), frailty was also associated with lower income. On the other hand, education had a remarkably positive linear effect on the social frailty domain. This result was surprising considering that in previous research the association of education with frailty was either non-significant (Gobbens et al., 2010d; Garcia-Garcia et al., 2011), or negative (Fried et al., 2001; Woo et al., 2005; Barreto Pde et al., 2012), with lower education levels predicting higher frailty. The present finding may be explained by different views and expectations of social support and relationship quality, from individuals with distinct education levels.

Death of a loved one was the only life event associated with frailty. Considering the well-documented physical and psychological impact of bereavement (Stroebe et al., 2007), it is understandable that this event could lead to frailty. Concomitantly, unhealthier lifestyle and dissatisfaction with living environment predicted frailty in general and in each domain. This provides further evidence of the previously described importance of health-related behavior (Avila-Funes et al., 2008; Fried et al., 2009; Gobbens et al., 2010d) and environmental factors (Hogan et al., 2003; Markle-Reid and Browne, 2003; Bergman et al., 2007) in precipitating frailty.

Self-reported comorbidity, as in previous research (Gobbens et al., 2010d), predicted frailty in general, as well as physical and psychological frailty. Most authors agree that comorbidity can lead to the onset of frailty (Bergman et al., 2007; Fried et al., 2009; Morley et al., 2013). Nonetheless, as described in other studies (Kriegsman et al., 1996), assessing comorbidity trough self-report may be susceptible to bias, mainly because of its dependence on the participants’ insight regarding chronic disease. Consequently, as it is directly linked with the amount of comorbidities, the number of daily-consumed drugs might have been a more precise indicator of the participants’ health status. Moreover, considering that self-reported comorbidity was rated dichotomously (yes/no), the assessment of the amount of consumed drugs not only is more accurate, but also allows a broader characterization of the heterogeneity of the participants’ comorbidity burden.

In fact, as hypothesized, the assessment of medication allowed the prediction of an additional variance of frailty, mainly because of the higher physical frailty of individuals who take greater amounts of medication. It can be discussed that assessing medication, a less subjective measure than self-reported comorbidity, was associated with the less subjective domain of frailty. Nonetheless, one should consider that these findings may also be linked with the adverse outcomes of polymedication and its association with frailty (Gnjidic et al., 2012a,b).

Frailty in general and physical frailty were also predicted by the consumption of medication for the cardiovascular system and for the blood and blood-forming organs. Considering that the latter medication type includes the antiplatelet drugs, which are the mainstay of cardiovascular disease prevention (Lin et al., 2010; Renda and de Caterina, 2012), it is possible to conclude that frailty was independently associated with the usage of drugs that target cardiovascular risk. Indeed, previous research (Fried et al., 2001; Afilalo et al., 2009; Solfrizzi et al., 2013) has shown that frailty – particularly physical frailty – may be associated with common cardiovascular diseases and with their own determinants.

Frailty was lower for those medicated for the cardiovascular system and higher for those who consumed antiplatelet drugs. Although there is limited evidence in this regard, one could argue that the drugs included in the first group were effective in minimizing cardiovascular risk factors and, therefore led to the prevention or to the decrease of frailty (Strandberg and Pitkala, 2007; Afilalo et al., 2009). Moreover, there are studies that suggest that ACE inhibitors improve physical function in elderly individuals, particularly regarding frailty related components, such as mobility and muscle strength (Hutcheon et al., 2002; Onder et al., 2002; Sumukadas et al., 2007). On the other hand, the results regarding the increased frailty of the participants who consumed antiplatelet drugs may be explained by the likely higher cardiovascular risk of these individuals. In fact, according to European Guidelines on Cardiovascular Disease Prevention in Clinical Practice (Perk et al., 2012), antiplatelet therapy, particularly low-dose of acetylsalicylic acid, should be prescribed to hypertensive patients with a history of cardiovascular events, with reduced renal function or with high cardiovascular risk. Furthermore, previous research (Burgess et al., 2007) has shown that the consumption of antithrombotics is associated with history of atrial fibrillation and stroke, which in turn can lead to frailty (Woods et al., 2005; Afilalo et al., 2009).

The main strengths of the present study are the statistical procedures used, the reinforcement of the current evidence supporting the multidimensional nature of frailty and of its predictors, and the findings regarding an ameliorated prediction of frailty based on an objective, easy to execute, assessment of medication. It is also the first study that analyzes the determinants considered in the integral conceptual model of frailty in elderly individuals from a southern European country. Some limitations of this study should be also noted. First, the non-probabilistic sampling method could have limited these findings namely in regard to their generalization. Moreover, in the present study, the proportion of women (75.8%) is considerably larger than the proportion of men. However, this difference roughly reflects the current sociodemographic characteristics of the Portuguese elderly population, in which there is an increasingly larger proportion of women in older age groups (INE, 2012). On the other hand, correlation coefficient values were somewhat low, probably due to the small sample size. Also, the cross-sectional design does not allow the examination of the temporal continuum between determinants, comorbidity, consumed medications and frailty, in order to conclude causality. In turn, the definition of broad medication types/groups could have limited the results. For example, the inclusion of antidiabetics as well as vitamins and mineral supplements in the same group could have limited the ability of this medication type to explain frailty variance. Taking this into consideration, as elderly individuals with diabetes tend to have a greater risk of becoming frail (Fried et al., 2001; Woods et al., 2005), an association would be expected if only antidiabetics were considered. Finally, the self-report nature of TFI can be considered a limitation because of its inherent subjectivity and reliance on memory and insight (Farias et al., 2005; Frank et al., 2011; Antoine et al., 2013). Nonetheless, TFI items correlated as expected with corresponding standardized measures in previous research (Gobbens et al., 2010e; Coelho et al., 2014) and, in the present study, the relationship between self-reported determinants and frailty was not significantly influenced by cognitive status.

Several directions for future research can be suggested. Longitudinal studies should be conducted to better examine how life course determinants and comorbidity predict frailty in the short, medium, and long term. Also, further studies should focus on the association of comorbidity and medications with the psychological and social domains of frailty. Likewise, the association between level of education and each frailty domain should be thoroughly analyzed, especially considering the findings of this study in regard to social frailty.

## Conclusion

This research provides important data about which factors may precipitate states of higher vulnerability in this elderly sample. Furthermore, the added value of a brief assessment of medication was significant, so it should be considered as supplementary to TFI. These findings should be taken into account for a more effective identification of frailty, and to implement timely and targeted interventions in order to treat this syndrome and prevent related adverse outcomes.

## Author Contributions

TC, CP and LF designed the study. TC was responsible for data collection, carried out the statistical analysis, and drafted the paper. CP and LF supervised the whole research and assisted with interpreting data and writing the article. RG provided advice and aided in the preparation of the final manuscript. All authors reviewed and provided valuable contributions to the whole manuscript.

## Conflict of Interest Statement

The authors declare that the research was conducted in the absence of any commercial or financial relationships that could be construed as a potential conflict of interest.
